# MiR-26a contributes to the PDGF-BB-induced phenotypic switch of vascular smooth muscle cells by suppressing Smad1

**DOI:** 10.18632/oncotarget.17998

**Published:** 2017-05-18

**Authors:** Xiaoyan Yang, Mei Dong, Hao Wen, Xiaoling Liu, Meng Zhang, Lianyue Ma, Cheng Zhang, Xiaorong Luan, Huixia Lu, Yun Zhang

**Affiliations:** ^1^ The Key Laboratory of Cardiovascular Remodeling and Function Research, Chinese Ministry of Education and Chinese Ministry of Health, Department of Cardiology, Shandong University Qilu Hospital, Jinan, Shandong, China; ^2^ Department of Cardiology, Beijing Chaoyang Hospital, Capital Medical University, Beijing, China

**Keywords:** miR-26a, vascular smooth muscle cells, PDGF-BB, phenotype, vascular disease

## Abstract

The phenotypic switch of vascular smooth muscle cells (VSMCs) is a key event in the pathogenesis of various vascular diseases, such as atherosclerosis and post-angioplasty restenosis. Small non-coding microRNAs (miRNAs) have emerged as critical modulators of VSMC function. In the present study, miR-26a was significantly increased in cultured VSMCs stimulated by platelet-derived growth factor-BB (PDGF-BB) and in arteries with neointimal lesion formation. Moreover, we demonstrated that miR-26a regulates the expression of VSMC differentiation marker genes such as α-smooth muscle actin (α-SMA), calponin and smooth muscle myosin heavy chain (SM-MHC) in PDGF-BB-treated VSMCs. We further confirmed that the regulatory effect of miR-26a during the phenotypic transition occurs through its target gene Smad1, which is a critical mediator of the pro-contractile signal transmitted by bone morphogenetic protein (BMP) and transforming growth factor-beta (TGF-β). This discovery proposed a new channel for communication between PDGF and the BMP/TGF-β family. We concluded that miR-26a is an important regulator in the PDGF-BB-mediated VSMC phenotypic transition by targeting Smad1. Interventions aimed at miR-26a may be promising in treating numerous proliferative vascular disorders.

## INTRODUCTION

Vascular smooth muscle cells (VSMCs) exhibit extensive plasticity and can undergo phenotypic changes from a quiescent contractile state to a proliferative synthetic state in response to various cellular stimuli [1, 2]. The aberrant transition of VSMCs phenotype plays a pivotal role in the pathogenesis of a variety of cardiovascular diseases, such as atherosclerosis, hypertension and postangioplasty restenosis [3, 4]. The synthetic VSMC phenotype is marked by increased migration, proliferation and production of extracellular matrix components as well as decreased expression of dedifferentiation markers, including α-smooth muscle actin (α-SMA), calponin and smooth muscle myosin heavy chains (SM-MHC) [1, 2]. The VSMC phenotype is modulated by a variety of environmental cues, including growth factors, cell-cell contact, extracellular matrix components, and neuronal input [2, 4]. Particularly, the phenotypic switch modulated by PDGF-BB has been thoroughly established and subsequently leads to the formation of neointima in response to vascular injury [5–7]. Therefore, understanding the molecular mechanism underlying PDGF-BB-induced VSMC phenotype modulation is a priority in this field.

MicroRNAs (miRNAs) are a class of small non-coding RNAs that posttrancriptionally regulate gene expression. MicroRNAs negatively control target gene expression through the blockage of target mRNA translation or the induction of target mRNA instability. MicroRNAs exhibit critical functions in a variety of biological processes [3, 4]. Several miRNAs have recently been identified as modulators of the VSMC phenotype *in vitro* and *in vivo*, including miR-145, miR-21, miR-221, miR-222, miR-146a and miR-663 [4, 7, 8]. Alterations in miRNA expression may have therapeutic potential in this context [7].

miR-26a is a highly conserved posttranscriptional regulator in various cellular processes, including proliferation, differentiation, migration and apoptosis. miR-26a was recently characterized as a tumor suppressor in hepatocellular carcinoma (HCC) by targeting multiple oncogenic proteins [9, 10]. Additionally, miR-26a plays important roles in cancers of the oral cavity, lung, cervix and bile duct [11–14]. In respiratory system, the study of miR-26a is primarily focused on pulmonary fibrosis. miR-26a is downregulated in mice with experimental pulmonary fibrosis [15] and exhibits anti-proliferative capacity in TGF-β1-induced human lung fibroblasts [16]. Moreover, miR-26a participates in the epithelial-mesenchymal transition (EMT) and promotes airway smooth muscle hypertrophy [17–19]. miR-26a overexpression inhibits endothelial cell tube formation and caudal vein plexus formation in zebrafish [20]. miR-26a expression is altered during abdominal aortic aneurysm (AAA) formation; thus, miR-26a was recently identified as a novel regulator of VSMC function [21]. The objective of this study was to determine whether miR-26a regulates the PDGF-BB-modulated VSMC phenotypic switch.

## RESULTS

### PDGF-BB alters the VSMC phenotype

**Figure 1:**
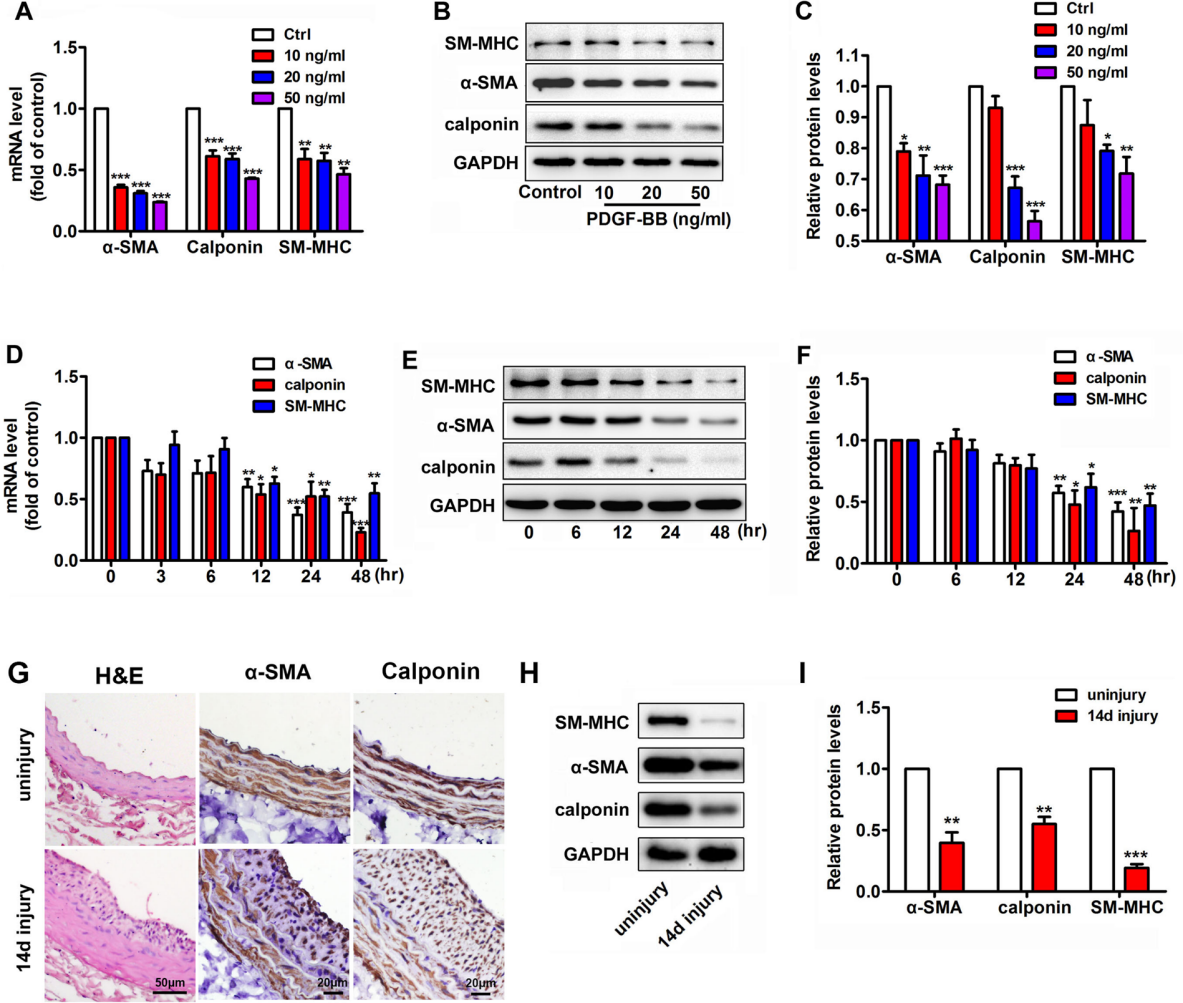
PDGF-BB dose- and time-dependent suppression of the expression of differentiation marker genes, such as α-SMA, calponin and SM-MHC, in murine vascular smooth muscle cells (VSMCs) (**A**) PDGF-BB induced a dose-dependent downregulation of VSMC differentiation marker genes, such as α-SMA, calponin and SM-MHC, at 24 h after PDGF-BB treatment as determined via qRT-PCR. (**B**) PDGF-BB caused a dose-dependent downregulation of VSMC differentiation marker genes at 24 h after PDGF-BB treatment, as determined via Western blot. (**C**) Quantitative Western blot analysis of differentiation marker genes in VSMCs stimulated by 0, 10, 20 and 50 ng/ml PDGF-BB. (**D**) PDGF-BB (20 ng/ml) induced a time-dependent decrease in VSMC differentiation marker genes as determined by qRT-PCR. (**E**) PDGF-BB (20 ng/ml) caused a time-dependent decrease in VSMC differentiation marker genes as determined by Western blot. (**F**) Quantitative Western blot analysis of differentiation marker gene expression in PDGF-BB (20 ng/ml) -stimulated VSMCs at various time points by Western blot. All cell experiments were repeated thrice and data are presented as mean ± SEM. *p < 0.05 vs control, **p < 0.01 vs control, ***p < 0.001 vs control. (**G**) Tissue sections of rat carotid arteries from 14 days after balloon injury and the uninjured control were stained with H&E, α-SMA, calponin stainning. (**H**) Representative Western blots of VSMC phenotype marker genes in rat carotid arteries 14 days after balloon injury compared with the uninjured control. (**I**) Quantitative analysis of differentiation marker gene expression in balloon-injured rat carotid arteries compared with the control (*n* = 5), data are presented as mean ± SEM. **p < 0.01 vs uninjury, ***p < 0.001 vs uninjury.

We previously demonstrated that PDGF-BB is upregulated in VSMCs in the balloon-injury model of rat [22], thereby confirming its key role in modulating the VSMC proliferation during neointimal hyperplasia. To observe the effect of PDGF-BB on VSMC phenotype transformation, murine VSMCs were treated with various concentrations of PDGF-BB for 24 h. PDGF-BB induced a dose-dependent suppression of the mRNA and protein levels of VSMC differentiation marker genes such as α-SMA, calponin and SM-MHC (Figure 1A–1C). To investigate the temporal effects of PDGF-BB on VSMC differentiation marker genes expression, VSMCs were stimulated with 20 ng/ml PDGF-BB for 0, 3, 6, 12, 24 and 48 h. α-SMA, calponin and SM-MHC mRNA were down-regulated in a time-dependent manner (Figure 1D). The protein levels of these genes in VSMCs after PDGF-BB treatment were consistent with the mRNA changes (Figure 1E and 1F). Compared with the uninjured controls, balloon-injured rat carotid arteries showed significant neointimal hyperplasia and accumulation of VSMCs after 14 days of balloon injury (Figure 1G). Interestingly, in the balloon-injured rat carotid arteries, both the mRNA and protein expression levels of VSMC differentiation marker genes were all significantly downregulated compared with the controls (Figure 1G–1I), thereby suggesting that the VSMCs within vascular walls went phenotypic change after injury.

### miR-26a expression is induced in rat carotid arteries after balloon injury and in PDGF-BB-treated VSMCs

**Figure 2:**
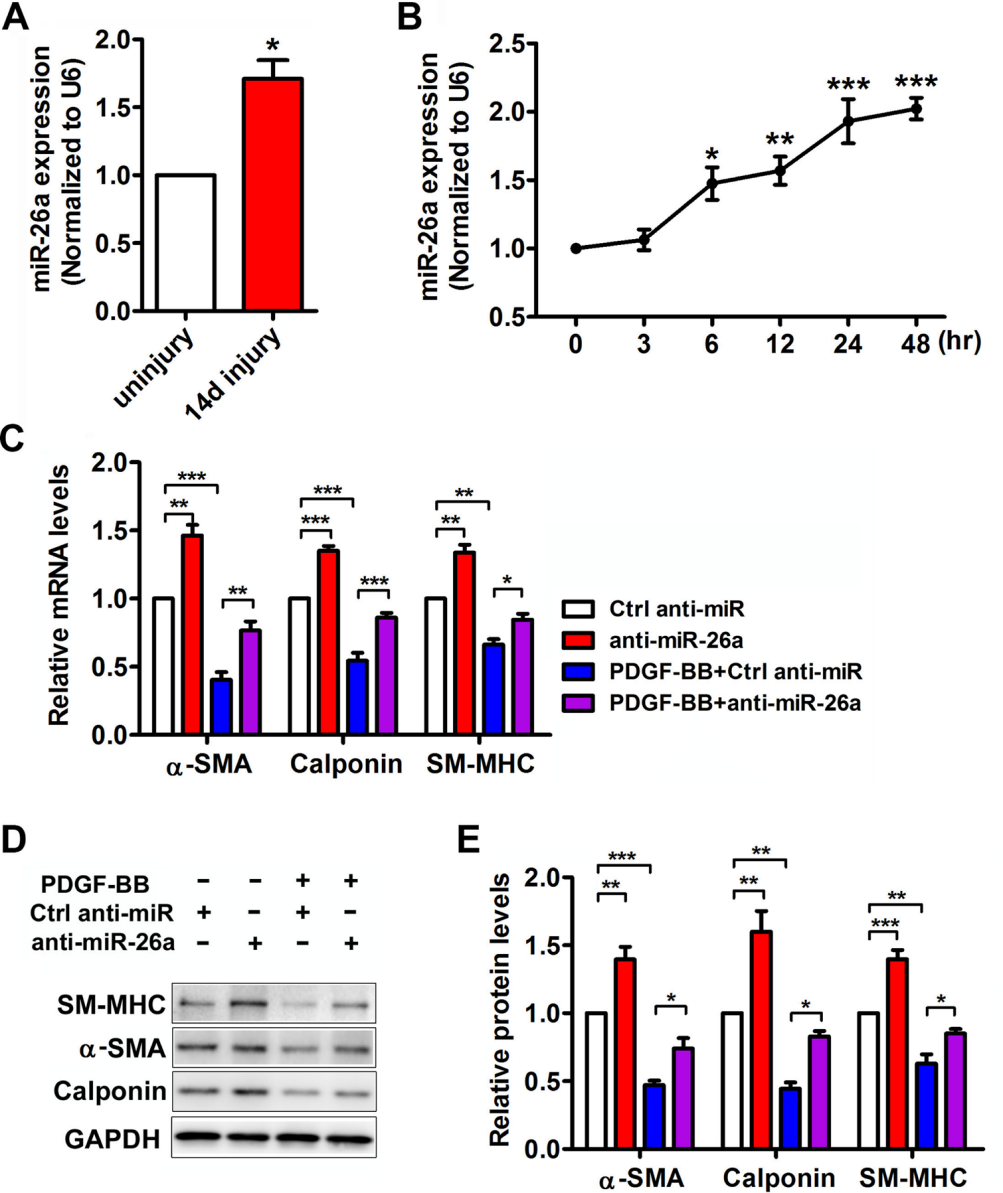
miR-26a is upregulated in balloon injured rat carotid arteries *in vivo* and in PDGF-BB-induced dedifferentiated VSMCs *in vitro* (**A**) miR-26a expression in rat carotid arteries 14 days after balloon injury and uninjured control as determined via qRT-PCR (*n* = 5), data are presented as mean ± SEM. *p < 0.05 vs uninjury. (**B**) miR-26a expression in VSMCs treated with PDGF-BB (20 ng/ml) at different time points as determined using qRT-PCR (*n* = 3), data are presented as mean ± SEM. *p < 0.05 vs 0 h, **p < 0.01 vs 0 h, ***p < 0.001 vs 0 h. VSMCs were pre-transfected with anti-miR-26a or ctrl anti-miR for 24 h followed by PDGF-BB treatment for 24 h. (**C**) anti-miR-26a suppressed PDGF-BB-mediated effects on VSMC phenotype marker genes as determined via qRT-PCR (*n* = 3), data are presented as mean ± SEM. *p < 0.05, **p < 0.01, ***p < 0.001. (**D**) anti-miR-26a abrogated PDGF-BB-mediated effects on VSMC phenotype marker genes as determined via Western blot. (**E**) Quantification analysis of VSMC phenotype marker genes in B (*n* = 3), data are presented as mean ± SEM. *p < 0.05, **p < 0.01, ***p < 0.001.

Previous studies indicated that miR-26a is involved in serum starvation-induced VSMC differentiation [21]. However, the role of miR-26a in PDGF-BB induced VSMC phenotypic modulation is unknown. Real-time PCR of miR-26a was performed both *in vivo* and *in vitro*. In the balloon-injured rat carotid arteries, the expression of miR-26a was notably increased compared with that of the control (Figure 2A). PDGF-BB significantly increased the miR-26a expression in VSMCs in a time-dependent manner (Figure 2B).

### miR-26a is a novel regulator in the PDGF-BB-mediated VSMC phenotypic switch

**Figure 3:**
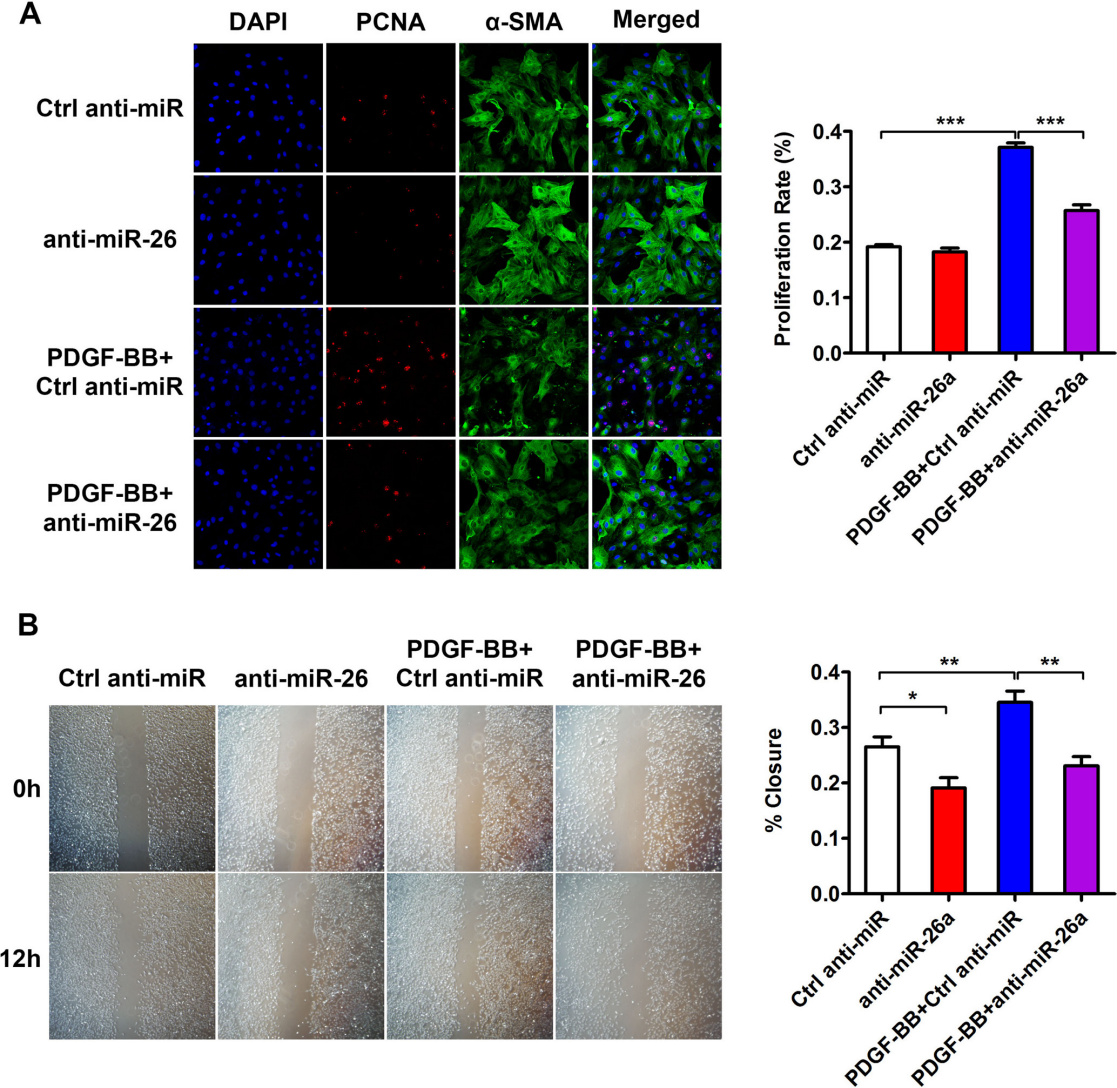
miR-26a is implicated in PDGF-BB-induced proliferation and migration in cultured VSMCs VSMCs were pre-transfected with anti-miR-26a or ctrl anti-miR for 24 h followed by PDGF-BB treatment for 24 h. (**A**) anti-miR-26a suppressed PDGF-BB-mediated effects on VSMC proliferation as determined via immunofluorescence staining with DAPI, PCNA, α-SMA and its quantification analysis (*n* = 3), all images were taken under the same magnification, data are presented as mean ± SEM. *p < 0.05, **p < 0.01, ***p < 0.001. (**B**) anti-miR-26a abrogated PDGF-BB-mediated effects on VSMC migration ability as determined via classic scratch assay and its quantification analysis (*n* = 5) all images were taken under the same magnification,, data are presented as mean ± SEM. *p < 0.05, **p < 0.01, ***p < 0.001.

To further investigate the role of miR-26a in the PDGF-BB-mediated VSMC phenotypic switch, we performed loss-of-function experiments by delivering a miR-26a inhibitor (anti-miR-26a) or control into VSMCs. 24 h after the transfection, the VSMCs were stimulated by PDGF-BB for another 24 h. The differentiation marker genes in VSMC significantly decreased after treatment with PDGF-BB. Transfection of anti-miR-26a partly abrogated the reduction of PDGF-BB on VSMC differentiation marker genes (Figure 2C–2E). PDGF-BB could significantly boost VSMC proliferation and migration, while inhibition of miR-26a significantly blunted PDGF-BB's effect on both proliferation and migration (Figure 3A and 3B). These results confirmed that miR-26a regulates PDGF-BB-mediated phenotypic changes in VSMCs.

### miR-26a is responsible for PDGF-BB-mediated decrease of Smad1 expression

**Figure 4:**
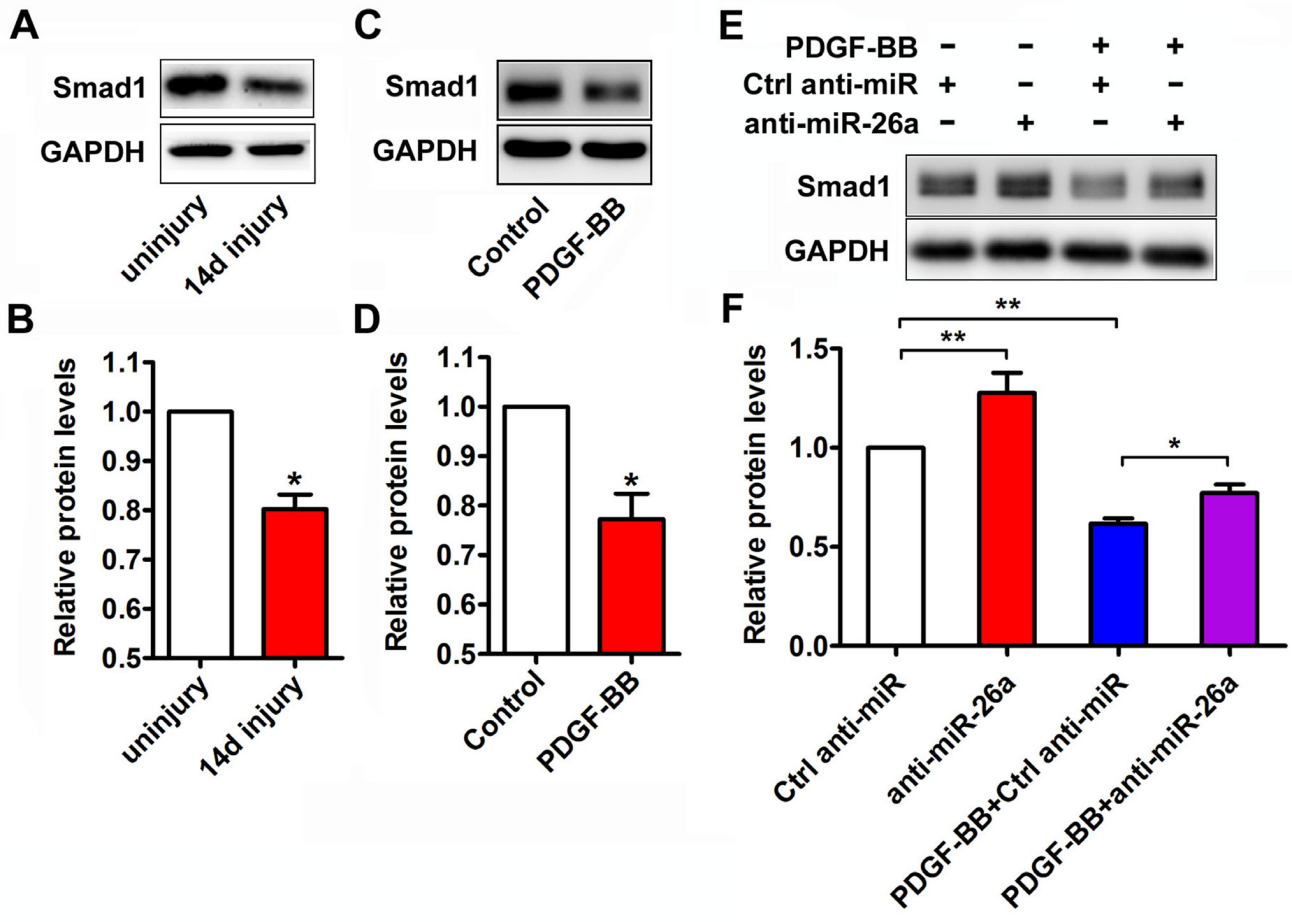
miR-26a modulated the expression of Smad1 during the PDGF-BB-induced phenotypic switch (**A**) Representative Western blots of Smad1 in rat carotid arteries 14 days after balloon injury compared with the uninjured control. (**B**) Quantification analysis of Smad1 expression in the injured vascular walls compared with uninjured controls as determined via Western blot (*n* = 5), data are presented as mean ± SEM. *p < 0.05 vs uninjured control. (**C**) Representative Western blots of Smad1 in VSMCs treated with PDGF-BB (20 ng/ml) for 24 h and vehicle control. (**D**) Quantification analysis of Smad1 expression in in VSMCs treated with PDGF-BB (20 ng/ml) for 24 h and control as determined via Western blot (*n* = 3), data are presented as mean ± SEM. *p < 0.05 vs control. (**E**) VSMCs were pre-transfected with anti-miR-26a or ctrl anti-miR for 24 h followed by PDGF-BB treatment for 24 h. Anti-miR-26a attenuated the PDGF-BB-mediated decrease of Smad1 as determined via Western blot. (**F**) Smad1 quantification analysis of VSMCs transfected with anti-miR-26a or ctrl anti-miR followed by PDGF-BB (20 ng/ml) treatment as determined by Western blot (*n* = 3), data are presented as mean ± SEM. *p < 0.05, **p < 0.01.

Smad1 is a critical signal transducer of the bone morphogenetic protein (BMP)/transforming growth factor-beta (TGFβ) pathway, which promotes a contractile phenotype [23]. miR-26a targets the 3′-UTR of Smad1 mRNA and downregulates Smad1 expression in VSMCs [20]. In this study, Smad1 protein expression was markedly reduced in balloon-injured rat arteries (Figure 4A and 4B) and in PDGF-BB treated VSMCs (Figure 4C and 4D). To investigate the role of miR-26a in the PDGF-BB-mediated Smad1 reduction, VSMCs were transfected with a miR-26a inhibitor or control before PDGF-BB stimulation. As expected, anti-miR-26a attenuated the inhibitory effect of PDGF-BB on Smad1 expression (Figure 4E and 4F). These results elucidate the regulatory effect of miR-26a on PDGF-BB-mediated Smad1 expression in VSMCs.

### Smad1 is involved in PDGF-BB-modulated VSMC phenotypic switch

**Figure 5:**
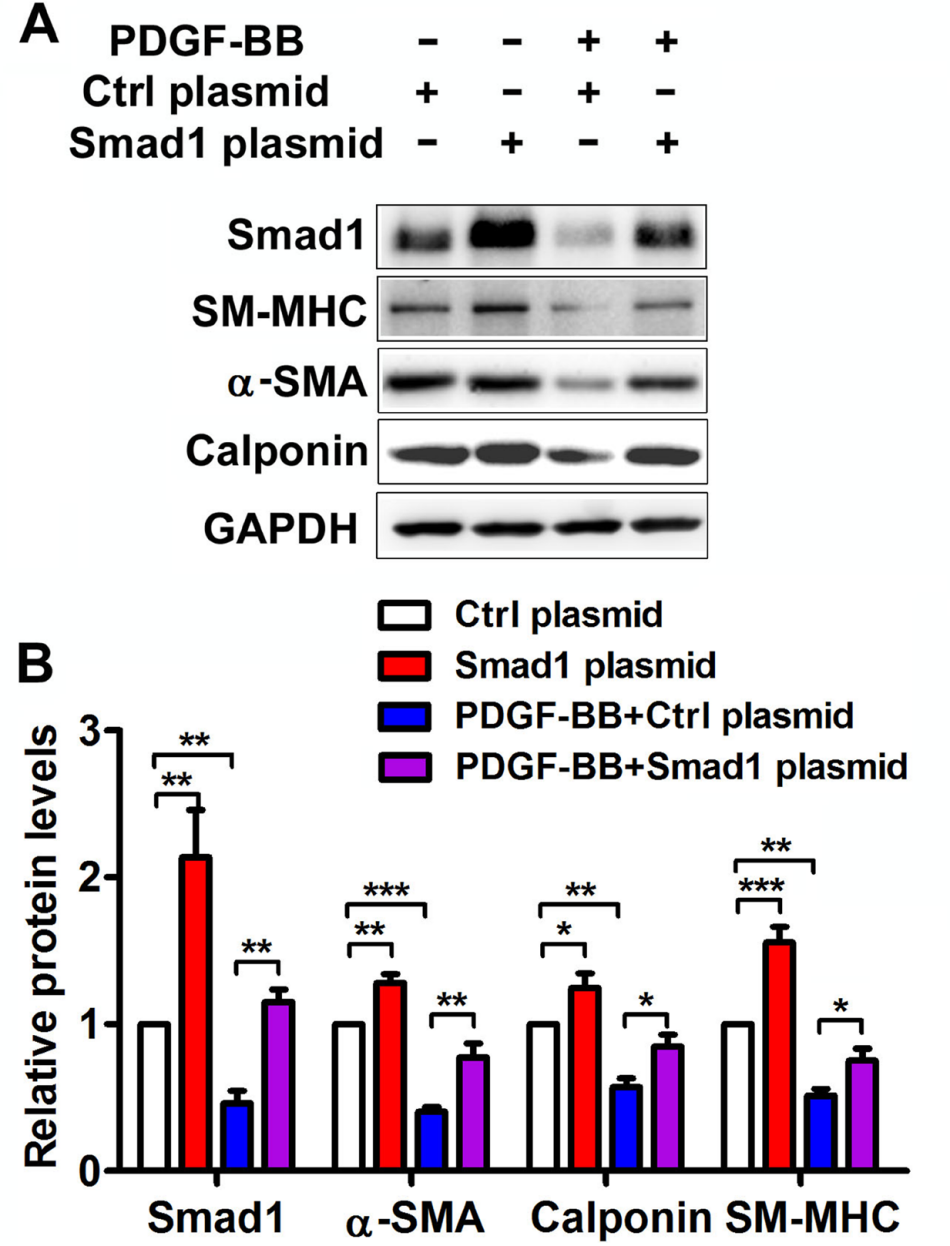
Smad1 is implicated in the PDGF-BB-mediated effects on VSMC phenotypic modulation The VSMCs were pre-transfected with Smad1 plasmid or Ctrl plasmid for 48 h followed by PDGF-BB treatment for 24 h. (**A**) Representative Western blots of phenotype marker genes and Smad1 in VSMCs transfected with Smad1 plasmid and Ctrl plasmid. (**B**) Quantification analysis of phenotype marker genes and Smad1 in VSMCs transfected with Smad1 plasmid and Ctrl plasmid (*n* = 3), data are presented as mean ± SEM. *p < 0.05. **p < 0.01, ***p < 0.001.

To determine the effects of Smad1 on the PDGF-BB-modulated VSMC phenotype, Smad1 was upregulated via a plasmid overexpressing Smad1 or downregulated via siRNA of Smad1. Interestingly, upregulation of Smad1 promotes the differentiation marker gene expression, including α-SMA, calponin and SM-MHC in VSMCs. Smad1 over-expression partly reversed the inhibitory action of PDGF-BB on differentiation marker in VSMCs (Figure 5A and 5B). More intriguingly, compared with anti-miR-26a group, Smad1 knockdown significantly downregulated the expression levels of α-SMA, calponin and SM-MHC, which means Smad1 knockdown could partially rescue the phenotypic change of VSMCs in anti-miR-26a transfected cells (Supplementary Figure 1). All these demonstrated that Smad1 is involved in PDGF-BB-modulated, miR-26a-participated VSMC phenotypic switch.

## DISCUSSION

VSMCs are highly versatile given the ever-changing environment of vascular walls. In a normal quiet state, the primary function of VSMCs involves contraction to sustain the blood flow. In response to local environmental changes, a series of modifications induced VSMCs to assume a proliferative, synthetic state. Phenotypic plasticity is essential for vascular development. However, aberrant phenotype switching contributes to the development of various proliferative vascular disorders. PDGF-BB is a pivotal modulator in the VSMC phenotype transformation [6]. As demonstrated in our previous studies, PDGF-BB is sharply elevated in the initiation stage during neointimal hyperplasia and promotes the accumulation of VSMCs after balloon injury [22]. However, the underlying molecular mechanisms remains unclear. In the present study, we demonstrate that miR-26a regulates the PDGF-BB-induced VSMC phenotypic switch by targeting Smad1.

miRNAs have recently been proven to play important roles in modulating VSMC phenotype [4, 7, 8]. For example, miR-145 is selectively expressed in the rodent VSMCs. It is significantly downregulated in the PDGF-BB-induced phenotypic changes *in vitro* and in balloon-injured rat carotid arteries. miR-145 deletion markedly compromises the VSMC contractile phenotype. Furthermore, restoration of miR-145 levels suppresses VSMC dedifferentiation and neointima formation after vascular injury by directly repressing the transcription factor KLF5 [7]. In this study, we explored that miR-26a expression was increased in balloon-injured rat carotid arteries and accompanied by VSMC phenotypic transformation within the vascular lesions with decreased α-SMA, calponin and SM-MHC expression. In cultured VSMCs treated with PDGF-BB, the expression variations of miR-26a were opposite those noted for the VSMC differentiation marker genes, such as α-SMA, calponin and SM-MHC. Taken together, non-coding RNA miR-26a potentially regulates PDGF-BB-induced VSMC phenotypic change. To elucidate the role of miR-26a in PDGF-BB-induced VSMC phenotypic modulation, we performed loss-of-function experiments. Anti-miR-26a increased the expression of differentiation marker genes, thereby suppressing the effect of PDGF-BB. These results revealed that miR-26a is a novel modulator for PDGF-BB induced VSMCs phenotype change.

Previous studies indicate that miR-26a binds to the 3′-UTR of Smad1 mRNA [20]. Whether Smad1 acts as a functional gene in the PDGF-BB-induced phenotypic transition remains unknown. We confirmed the negative relationship between miR-26a expression and Smad1 expression in both PDGF-BB-stimulated VSMCs and balloon-injured carotid arteries. Moreover, we found that miR-26a was capable of regulating Smad1 expression in PDGF-BB stimulated VSMCs. In addition, overexpression of Smad1 abrogates the inhibitory effect of PDGF-BB on VSMC contractile phenotype. On the other hand, Smad1 knockdown could partially rescue the phenotypic change of VSMCs in anti-miR-26a transfected cells. These results indicated that Smad1 is possibly responsible for miR-26a-mediated effects on the VSMC phenotypic shifts induced by PDGF-BB .

BMP and TGFβ signalling pathways were able to promote the contractile phenotype. Smad proteins are critical mediators of the pro-contractile signal transmitted by BMP and TGFβ [23, 24]. A recent study reported that PDGF-BB induces miR-24 expression and degrades of the Trb3 mRNA, subsequently leading to the downregulation of Smad signal transducers [25]. This molecular mechanism provides new insight into the antagonistic effect between PDGF-BB and the TGFβ family in establishing the VSMC phenotype. The molecular mechanisms underlying the regulation of the VSMC phenotype in previously discovered miRNAs, such as miR-143/145, miR-31, miR-221 and miR-146a were primarily involved in the direct or indirect regulation of the expression of transcriptional factors, including serum response factor, myocardin and myocardin-related transcription factors (MRTFs) as well as the Kruppel-like zinc finger family [3, 4, 7, 26]. In the present study, we identified miR-26a as another potential mediator of the relationship between PDGF and the TGFβ family. miR-26a targets the effector molecule Smad1 of the BMP/TGFβ signaling pathway.

We primarily focused on the role of miR-26a in the PDGF-BB induced VSMC phenotype transition. This study did not establish the molecular mechanisms underlying miR-26a-mediated interactions between PDGF-BB and TGFβ family. In addition, miR-26a should be genetically manipulated in further studies using balloon-injured rat models to determine whether neointimal hyperplasia can be attenuated in this scenario.

**Figure 6:**
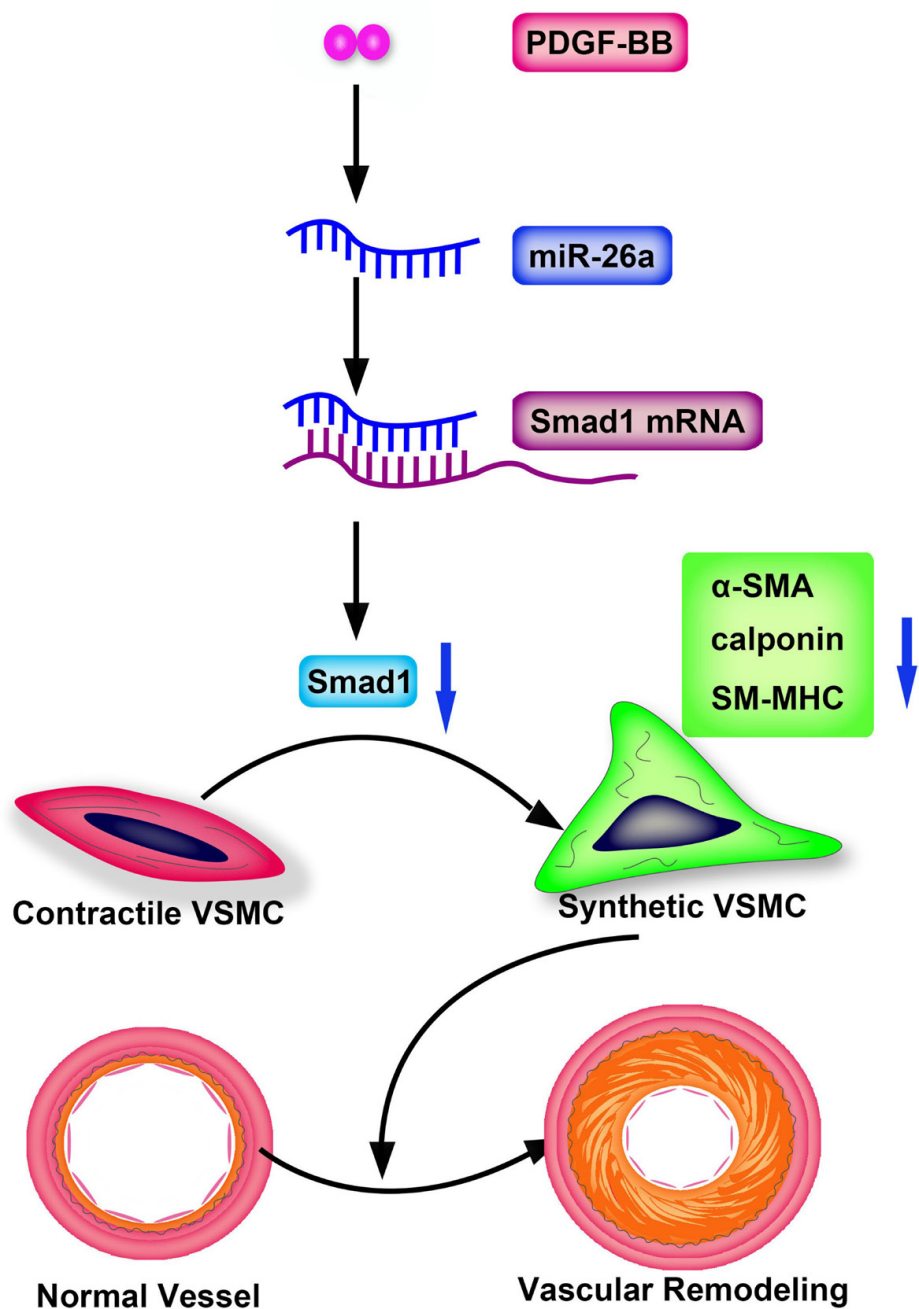
Schematic representation of miR-26a in the PDGF-BB–mediated phenotypic switch of VSMC PDGF-BB increases the miR-26a level that leads to suppression of Smad1. The downregulated Smad1 causes VSMC switching to a synthetic phenotype and eventually leads to vascular remodeling.

In summary, we identified miR-26a as a crucial regulator in the PDGF-BB induced phenotype switch of VSMC by targeting, at least partially, the Smad1 pathway. Vascular injury and PDGF-BB are capable of increasing miR-26a levels in VSMCs. Increased miR-26a expression could reduce the expression of its target gene Smad1. Smad1 overexpression could partially reverse the PDGF-BB-modulate VSMC differentiation marker genes expression, restoring a contractile VSMC phenotype (Figure 6). These findings suggested that the PDGF-BB-miR-26a-smad1 pathway may serve as a potential therapeutic target in the phenotypic switch of VSMCs.

## MATERIALS AND METHODS

### Animals

All animal studies were approved by the Ethical Committee of Shandong University Qilu Hospital.

### Reagents and antibodies

PDGF-BB was purchased from PeproTech (Rocky Hill, USA). The following antibodies were used: rabbit anti-alpha smooth muscle actin (α-SMA), rabbit anti-smooth muscle myosin heavy chain (SM-MHC), rabbit anti-calponin (Abcam, Cambridge,UK), rabbit anti-Smad1 (Cell Signaling Technology, Beverly, MA, USA) and rabbit anti-GAPDH (Sigma, St. Louis, USA). miR-26a inhibitor, Smad1 plasmids and siRNA were from GenePharma (Shanghai, China). TaqMan^®^ microRNA assays were purchased from Ambion (Carlsbad, USA). TRIzol^®^ reagent, Lipofectamine^TM^ RNAiMAX and Lipofectamine^®^ LTX and Plus™ Reagent were purchased from Invitrogen (Carlsbad, USA).

### Cell culture

Primary mouse aortic vascular smooth muscle cells were obtained from CHI Scientific (Jiangyin, China) and maintained in Dulbecco's modified Eagle's medium (DMEM) containing 10% FBS (Invitrogen, Carlsbad, CA, USA) in a 5% CO_2_ humidified incubator at 37°C. Cells passed less than 8 times were used for all the experiments.

### Rat carotid artery balloon injury model

Carotid artery balloon injury was performed in male Sprague-Dawley rats weighing 400 to 450 grams as described [27]. Ten male Sprague-Dawley rats were housed under constant room temperature (22°C) and ad libitum fed with a normal diet. All rats underwent anesthesia with pentobarbital (ip. 30 mg/kg) and left carotid artery was exposed. Then carefully insert an uninflated 2 French arterial balloon embolectomy catheter (Abbott Vascular) through the arteriotomy and down the entire length of the common carotid artery to the aortic arch. The balloon catheter was slowly inflated with 20 ul of saline and went back and forth three times to induce endothelial injury in injury group. While the right carotid arteries were used as uninjury control. After another 14 days, both carotid arteries were harvested, and were snap frozen in liquid nitrogen and stored at –80°C for western-blotting analysis, or fixed with 4% formaldehyde for immuno-histochemical analysis.

### Oligonucleotide transfection and overexpression of Smad1 in cultured VSMCs

For miR-26a knockdown, the miR-26a inhibitor (GenePharma), also known as anti-miR-26a, or the control was added to the complexes at a final concentration of 80 nmol/L. For Smad1 knockdown, the siRNA (GenePharma) or the control was added to the complexes at a final concentration of 40 nmol/L. Sequences of siRNA (5′-3′) are: CAGGCGACAUAUUGGGAAATT; UUUCCCAAU AUGUCGCCUGTT. For Smad1 overexpression, the Smad1 plasmid (GenePharma) was applied. Cells were transfected using Lipofectamine^TM^ RNAiMAX (for oligonucleotides) or Lipofectamine^®^ LTX and Plus™ Reagent (for plasmids) according to the manufacturer's protocol.

### Polymerase chain reaction analysis and RNA analysis via quantitative real-time polymerase chain reaction

Total RNA was extracted from murine aortic VSMCs or rat carotid arteries via TRIzol^®^ Reagent according to the manufacturer's protocol. Real-time reverse transcription polymerase chain reaction (qRT-PCR) of miR-26a and U6 were performed using TaqMan^®^ microRNA assays according to the manufacturer's protocol. Primer sequences (5′–3′) used for the detection of α-SMA, calponin, and SM-MHC are as follows: α-SMA Forward: GTCCCAGACATCAGGGAGTAA; Reverse: TCGGATACTTCAGCGTCAGGA; Calponin Forward: TCTGCACATTTTAACCGAGGTC; Reverse: GCCAG CTTGTTCTTTACTTCAGC; SM-MHC Forward: AAG CTGCGGCTAGAGGTCA; Reverse: CCCTCCCTTTG ATGGCTGAG; The expression of miR-26a relative to U6 and the expression levels of α-SMA, calponin, and SM-MHC relative to GAPDH were determined using the 2^–ΔΔCt^ method.

### Western blot

Standard western blot analysis was conducted. Briefly, cell or tissue lyses were resolved via sodium dodecyl sulphate polyacrylamide gel electrophoresis (SDS-PAGE) and transferred to polyvinylidene fluoride (PVDF) membranes. Blots were blocked with 5% nonfat milk in tris-buffered saline (TBS) with 0.1% Tween20 and developed with diluted antibodies to α-SMA (1:1,000 dilution), Smad1 antibody (1:1,000 dilution), SM-MHC (1:1,000 dilution), calponin (1:1,000 dilution), and GAPDH (1:5,000 dilution). The blots were then incubated with secondary antibodies and visualized using ECL Prime Western blotting detection reagent from GE Healthcare (Pittsburgh, PA, USA).

### Statistical analysis

Data are presented as means ± standard error of the mean (SEM). Differences between two groups were analysed with an unpaired *t*-test, and comparisons of more than two groups were performed with one-way analysis of variance (ANOVA) as appropriate. *P* < 0.05 was considered statistically significant.

## SUPPLEMENTARY MATERIALS FIGURE


