# Supplementary material for: MiR-26a contributes to the PDGF-BB-induced phenotypic switch of vascular smooth muscle cells by suppressing Smad1
Source: Oncotarget. 2017 May 18;8(44):75844–53. doi: 10.18632/oncotarget.17998 (PMC5652667; doi:10.18632/oncotarget.17998)
Supplement: Supplementary file 1 [file oncotarget-08-75844-s001.pdf]

MiR-26a contributes to the PDGF-BB-induced phenotypic switch of vascular smooth muscle cells by suppressing Smad1

SUPPLEMENTARY MATERIALS

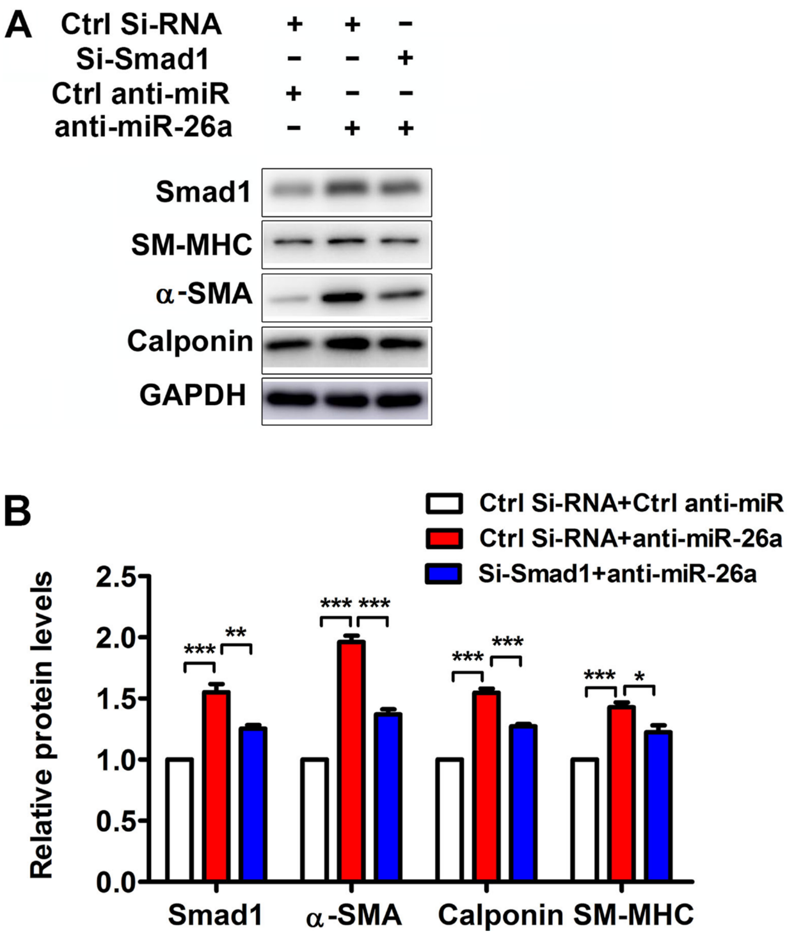

Supplementary Figure 1: Smad1 knockdown could partially rescue the phenotypic change of VSMCs in anti-miR-26a transfected cells. The VSMCs were pre-transfected with Si-Smad1or Ctrl siRNA for 48 h followed by anti-miR26a treatment for 24 h. (**A**) Representative Western blots of phenotype marker genes and Smad1 in VSMCs transfected with Si-Smad1, Ctrl siRNA and anti-miR 26a. (**B**) Quantification analysis of phenotype marker genes and Smad1 in VSMCs transfected with Si-Smad1, Ctrl siRNA and anti-miR 26a ($n = 3$), data are presented as mean ± SEM. *$p < 0.05$. **$p < 0.01$, ***$p < 0.001$.
